# Bi-allelic variants in *MRPL49* cause variable clinical presentations, including sensorineural hearing loss, leukodystrophy, and ovarian insufficiency

**DOI:** 10.1016/j.ajhg.2025.02.005

**Published:** 2025-03-04

**Authors:** Huw B. Thomas, Leigh A.M. Demain, Alfredo Cabrera-Orefice, Isabelle Schrauwen, Hanan E. Shamseldin, Alessandro Rea, Thashi Bharadwaj, Thomas B. Smith, Monika Oláhová, Kyle Thompson, Langping He, Namanpreet Kaur, Anju Shukla, Musaad Abukhalid, Muhammad Ansar, Sakina Rehman, Saima Riazuddin, Firdous Abdulwahab, Janine M. Smith, Zornitza Stark, Hanifenur Mancilar, Sait Tumer, Fatma N. Esen, Eyyup Uctepe, Vehap Topcu, Ahmet Yesilyurt, Erum Afzal, Mehri Salari, Christopher Carroll, Giovanni Zifarelli, Peter Bauer, Deniz Kor, Fatma D. Bulut, Henry Houlden, Reza Maroofian, Samantha Carrera, Wyatt W. Yue, Kevin J. Munro, Fowzan S. Alkuraya, Peter Jamieson, Zubair M. Ahmed, Suzanne M. Leal, Robert W. Taylor, Ilka Wittig, Raymond T. O’Keefe, William G. Newman

**Affiliations:** 1 Division of Evolution, Infection and Genomics, School of Biological Sciences, University of Manchester, Manchester M13 9PL, UK; 2 Manchester Centre for Genomic Medicine, St Mary’s Hospital, Manchester University NHS Foundation Trust, Manchester M13 9WL, UK; 3 Centre for Functional Proteomics, Institute for Cardiovascular Physiology, Medical Faculty, Goethe University, 60596 Frankfurt am Main, Germany; 4 Institute of Biochemistry, Medical Faculty, Justus-Liebig-University, 35392 Giessen, Germany; 5 Department of Translational Neurosciences, University of Arizona College of Medicine Phoenix, Phoenix, AZ, USA; 6 Department of Translational Genomics, Center for Genomic Medicine, King Faisal Specialist Hospital and Research Center, Riyadh, Saudi Arabia; 7 Center for Statistical Genetics, Department of Neurology, Gertrude H. Sergievsky Center, Columbia University Medical Center, New York, NY 10032, USA; 8 Department of Applied Sciences, Faculty of Health & Life Sciences, Northumbria University, Newcastle upon Tyne, UK; 9 Mitochondrial Research Group, Clinical and Translational Research Institute, Faculty of Medical Sciences, Newcastle University, Newcastle upon Tyne NE2 4HH, UK; 10 NHS Highly Specialised Service for Rare Mitochondrial Disorders, Newcastle upon Tyne Hospitals NHS Foundation Trust, Newcastle upon Tyne NE1 4LP, UK; 11 Department of Medical Genetics, Kasturba Medical College Manipal, Manipal Academy of Higher Education, Manipal, Karnataka, India; 12 Department of Neuroscience, King Faisal Specialist Hospital and Research Center, Riyadh, Saudi Arabia; 13 Department of Biochemistry, Faculty of Biological Sciences, Quaid-I-Azam University, Islamabad 45320, Pakistan; 14 Department of Otorhinolaryngology - Head & Neck Surgery, School of Medicine University of Maryland, Baltimore, MD, USA; 15 Centre of Excellence in Molecular Biology, University of the Punjab, Lahore, Pakistan; 16 Specialty of Genomic Medicine, Faculty of Medicine and Health, University of Sydney, Sydney, NSW 2000, Australia; 17 Western Sydney Genetics Program, Department of Clinical Genetics, Sydney Children’s Hospitals Network, Westmead, NSW 2145, Australia; 18 Victorian Clinical Genetics Services, Murdoch Children’s Research Institute, Flemington Road, Melbourne, VIC, Australia; 19 University of Melbourne, Melbourne, VIC, Australia; 20 Acıbadem Labgen Genetic Diagnosis Center, Istanbul, Türkiye; 21 Department of Development Pediatrics, The Children’s Hospital and The Institute of Child Health, Multan, Pakistan; 22 Department of Neurology, Shahid Beheshti University of Medical Sciences, Tehran, Iran; 23 Genetics Section, Molecular and Clinical Sciences Research Institute, St. George’s, University of London, London, UK; 24 CENTOGENE GmbH, Am Strande 7, 18055 Rostock, Germany; 25 Cukurova University, Medical Faculty, Department of Pediatric Metabolism and Nutrition, Adana, Turkey; 26 Department of Neuromuscular Diseases, University College London, Queen Square, Institute of Neurology, London WC1N 3BG, UK; 27 Genome Editing Unit Core Facility, Faculty of Biology, Medicine and Health, University of Manchester, Manchester, UK; 28 Newcastle University Biosciences Institute, Medical School, Framlington Place, Newcastle upon Tyne NE2 4HH, UK; 29 Manchester Centre for Audiology and Deafness (ManCAD), School of Health Sciences, University of Manchester, Manchester, UK; 30 Department of Anatomy and Cell Biology, College of Medicine, Alfaisal University, Riyadh, Saudi Arabia; 31 Department of Radiology, Manchester University Hospital NHS Foundation Trust, Manchester M13 9PW, UK; 32 Taub Institute for Alzheimer’s Disease and the Aging Brain, and the Department of Neurology, Columbia University Medical Center, New York, NY, USA; 33 German Center for Cardiovascular Research (DZHK), Partner Site Rhein Main, 60596 Frankfurt am Main, Germany

**Keywords:** MRPL49, mitoribosome, Perrault syndrome, sensorineural hearing loss, primary ovarian insufficiency, leukodystrophy, rare disease, mitochondria, combined oxidative phosphorylation deficiency, learning disability

## Abstract

Combined oxidative phosphorylation deficiency (COXPD) is a rare multisystem disorder that is clinically and genetically heterogeneous. Genome sequencing identified bi-allelic *MRPL49* variants in individuals from nine unrelated families with presentations ranging from Perrault syndrome (primary ovarian insufficiency and sensorineural hearing loss) to severe childhood onset of leukodystrophy, learning disability, microcephaly, and retinal dystrophy. Complexome profiling of fibroblasts from affected individuals revealed reduced levels of the small mitochondrial ribosomal subunits and a more pronounced reduction of the large mitochondrial ribosomal subunits. There was no evidence of altered mitoribosomal assembly. The reductions in levels of oxidative phosphorylation (OXPHOS) enzyme complexes I and IV are consistent with a form of COXPD associated with bi-allelic *MRPL49* variants, expanding the understanding of how disruption of the mitochondrial ribosomal large subunit results in multisystem phenotypes.

## Graphical abstract

**Figure:**
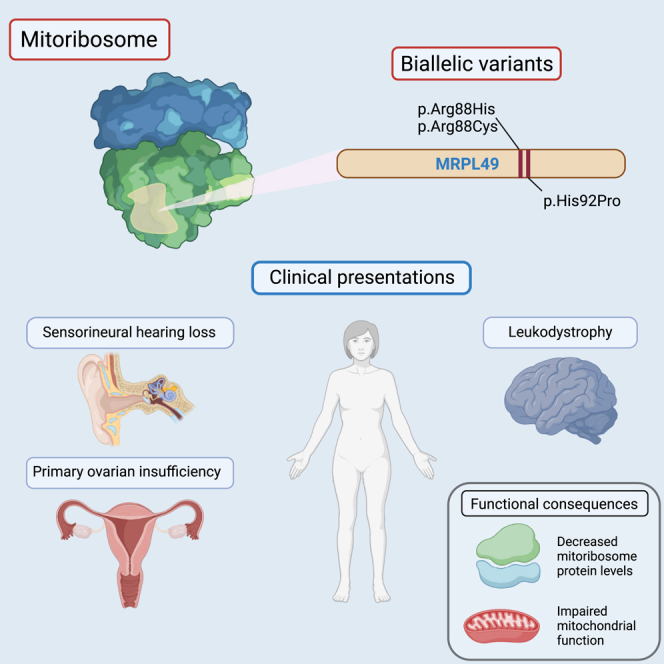

## Teaser

We identify bi-allelic variants in the mitoribosomal large subunit encoded by *MRPL49* as a cause of a pleiotropic presentation of hearing loss, ovarian failure, learning disability, and leukodystrophy and support these findings with genetic and functional data.

## Main text

The mitochondrial ribosome (mitoribosome) is a 55S ribonucleoprotein complex composed of large and small subunits that coordinates the synthesis of the 13 proteins coded by the mitochondrial genome. These 13 proteins are vital components of the oxidative phosphorylation (OXPHOS) enzyme complexes. Human mitoribosomes are tethered to the mitochondrial inner membrane through the 39S large subunit of the mitoribosome.^1^ This large subunit (mt-LSU) is comprised of two structural RNA molecules, a 16S ribosomal RNA (rRNA) and mt-tRNA^Val^, and 52 proteins.^2^^,^^3^^,^^4^^,^^5^ In contrast, the 28S small subunit (mt-SSU) is comprised of 30 proteins and a 12S rRNA.^6^ Several human diseases are caused by germline variants in genes encoding mitoribosome proteins.^5^^,^^7^ However, despite the number of mitoribosome large subunit proteins, bi-allelic pathogenic variants have been identified in genes encoding only a limited amount (Table S1). Affected individuals with pathogenic variants in mt-LSU genes have a broad range of clinical presentations encompassing cardiomyopathy, liver dysfunction, neurological phenotypes, and ovarian insufficiency.^8^^,^^9^^,^^10^^,^^11^^,^^12^ In all reported individuals with disorders due to variants in genes encoding the mt-LSU, with the exception of *MRPL50*, there were measurable defects in mitochondrial OXPHOS, consistent with the definition of combined oxidative phosphorylation deficiency (COXPD; MIM: 609060) disorders.

Here, we report on individuals from nine unrelated families with bi-allelic variants in *MRPL49* (MIM: 606866) characterized by diverse clinical phenotypes encompassing bilateral sensorineural hearing loss (SNHL) and primary ovarian insufficiency (POI; Perrault syndrome, MIM: 233400), microcephaly, learning disability, developmental delay, leukodystrophy, and retinal disease. Informed consent for diagnostic and research studies was obtained for all subjects in accordance with Declaration of Helsinki protocols and approved by local institutional review boards. Ethical approval for this study was granted by the National Health Service Ethics Committee (16/WA/0017) and the University of Manchester.

**Figure 1:**
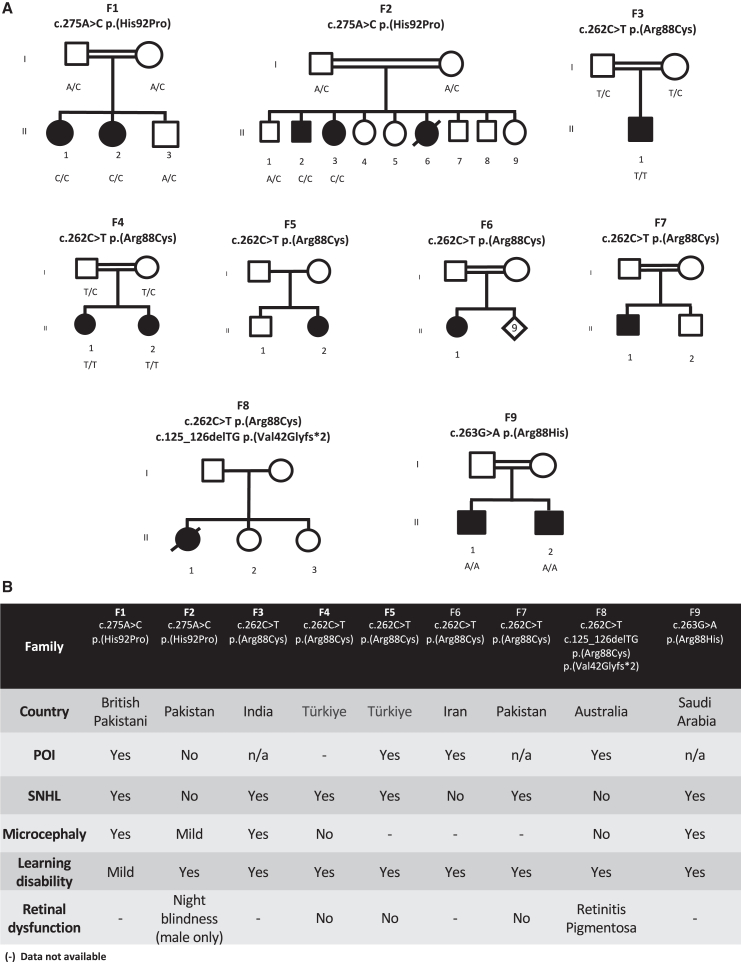
Pedigree and clinical data for families with bi-allelic *MRPL49* variants (A) Pedigree information for nine unrelated families of individuals with either homozygous or compound heterozygous variants in *MRPL49* (GenBank: NM_004927.4) and (B) accompanying clinical details of POI, SNHL, and learning disability shared across probands.

A young British Pakistani woman and her sister (family 1 [F1]) were referred with a possible diagnosis of Perrault syndrome (Figure 1A). The proband was 16 years old with bilateral, high-frequency, profound SNHL (Figure S1), initially diagnosed at 4 years of age. She had absent menarche. Biochemical analysis revealed raised gonadotrophins (luteinizing hormone [LH]: 24 IU/L [normal range (N): 5–25], follicle-stimulating hormone [FSH]: 109 IU/L [N: 0.3–10], and decreased estradiol <37 pg/mL [N: 37–400]), consistent with hypergonadotropic hypogonadism. Pelvic imaging revealed absent ovaries, vagina, uterus, and cervix. She was commenced on ethinylestradiol replacement treatment. She was of normal height and weight but noted to have microcephaly (occipital frontal circumference [OFC]: 50.6 cm, −3.5 SD) and non-progressive mild learning disability. Her sister was diagnosed with SNHL at age 4 years. She also had mild learning and behavioral problems and microcephaly (OFC: 48.7 cm, −3.7 SD). At age 12 years, she had normal height and weight parameters and raised gonadotrophins (LH: 42.1 IU/L and FSH: 118 IU/L), consistent with hypergonadotropic hypogonadism. Pelvic imaging revealed a small uterus and absent ovaries (Figure S3). She has been commenced on estradiol.

Exome sequencing did not identify variants in any of the known Perrault syndrome genes^13^^,^^14^ or genes associated with SNHL or POI in these two affected individuals. Autozygosity mapping using SNP arrays identified 11 homozygous regions >2 Mb. Within the largest region of autozygosity on chromosome 11 (∼50 Mb), a rare homozygous variant in *MRPL49* (c.275A>C [GenBank: NM_004927.4]; p.His92Pro) (Figure 3A) was identified in both sisters. The variant was heterozygous in both unaffected parents and a clinically unaffected brother. Through Genematcher^15^ and international collaboration, we identified eight additional families, including individuals presenting with bi-allelic variants in *MRPL49* displaying variable clinical presentations (Figure 1B).

In F2, the same missense variant as identified in F1, *MRPL49* c.275A>C (p.His92Pro), was identified in a brother and sister from a consanguineous family affected by intellectual disability and facial dysmorphism. It was not possible to formally assess the hearing status as the affected individuals were unable to undergo testing, but there was no evidence of a hearing deficit. The female (F2:II-3) had no overt reproductive abnormalities, with a normal menstrual cycle. The affected male (F2:II-2) had night blindness, bone abnormalities, borderline short stature (−1.8 SD), and seizures. He had borderline microcephaly at the time of initial assessment, but his current head circumference is within the normal range. This family was also of Pakistani ancestry, and haplotype analysis of exomes from affected individuals in F1 and F2 revealed a shared haplotype of 1.24 Mb (chr11:64,343,482–65,583,893 [GRCh38]), indicating a common ancestor founder variant.

In a consanguineous Indian family (F3), a homozygous (c.262C>T [GenBank: NM_004927.4] [p.Arg88Cys]) variant in *MRPL49* was identified in a 5-year-old boy with hearing loss. He was born at term with a birth weight of 2.25 kg (−3.37 SD). He had delayed attainment of age-appropriate milestones, standing only with support by 3 years and 6 months. At 5 years old, he could speak only monosyllables. He had episodes of fever associated with a loss of consciousness that were treated with anticonvulsants. At 5 years of age, his weight was 12.54 kg (−2.6 SD), his height was 103 cm with contractures (−1.6 SD), and his head circumference was 47 cm (−3.2 SD). He had a small forehead, plagiocephaly, low anterior hairline, metopic prominence, strabismus, and orofacial dystonia. He exhibited hypertonia (lower limbs > upper limbs) and tremors.

In four further families, we identified the same homozygous c.262C>T (p.Arg88Cys) variant. F4 is a consanguineous Turkish family of two affected sisters. The proband (F4:II-2) was diagnosed at 3 years of age with bilateral SNHL, for which she wears bilateral hearing aids. At age 5 years, she has moderate learning disability, strabismus, a height of 99 cm (−1.89 SD), and a head circumference of 49 cm (−0.94 SD). Her elder sister (F4:II-1) was diagnosed at age 6 years with profound SNHL. She has a unilateral cochlear implant and wears one hearing aid. At age 9, she is 119 cm tall (−2.3 SD) and has a head circumference of 51 cm (−0.7 SD). She had a febrile seizure at 2 years of age. They have no other siblings.

In F5 is a 12-year-old Turkish girl (F5:II-2) born to parents from the same village who are not known to be related to each other. She presented at 2 days old with hypoglycemia (blood glucose: 27 mg/dL [normal: >40 mg/dL]). At 6.5 years of age, she was noted to have severe hearing loss in her right ear with profound SNHL in her left ear. Her height is 141 cm (−2.15 SD). At 12 years of age, she has not undergone menarche and has biochemical evidence of hypergonadotrophic hypogonadism (FSH: 129.42 IU/L, LH: 23.52 IU/L, and estradiol < 15 pg/mL). She has an unaffected brother.

F6 comprises a 33-year-old woman (F6:II-1) from a consanguineous Iranian family. She has no hearing impairment but primary amenorrhea and moderate intellectual disability. Her height is 146 cm (−2.66 SD). She has 9 unaffected siblings. F7 comprises a 14-year-old male (F7:II-1) from a consanguineous Pakistani family. He was diagnosed at 14 years with moderate to severe bilateral SNHL treated with bilateral hearing aids. He has had moderate intellectual disability and has had episodes of lactic acidosis. His height is 169 cm (0.67 SD). He has an unaffected younger brother.

Of note, for a 17-year-old Turkish female with intellectual disability, bilateral SNHL requiring cochlear implants, kyphoscoliosis, and POI, a previous study suggested that the molecular explanation was a homozygous variant in *PANX1*.^16^ She was also homozygous for the same p.Arg88Cys *MRPL49* missense variant as identified in F3, F4, F5, F6, and F7. As no further individuals with bi-allelic *PANX1* variants and a similar phenotype have been reported, and since *PANX1* variants were not identified in any individual in our cohort and this alternative explanation exists, we are confident that the homozygous p.Arg88Cys *MRPL49* variant explains her clinical presentation.

In F8, an Australian woman (of Northern European ancestry) was identified in whom trio genome sequencing revealed that she was a compound heterozygote for the same missense variant c.262C>T (p.Arg88Cys) identified in F3-7 in *trans* to a predicted loss-of-function frameshift variant (c.125_126delTG [GenBank: NM_004927.4] [p.Val42Glyfs^∗^2]) in *MRPL49*. She was referred at the age of 37 years for a clinical genetics assessment to consider an underlying genetic cause for her intellectual disability, epilepsy, retinitis pigmentosa, bilateral posterior subcapsular cataracts, obesity, hypothyroidism treated with thyroxine, menstrual cycle anomalies, and recent psychosis with neurocognitive decline. There was no history of SNHL. The proband is one of three children born to unrelated parents. There are also two maternal half-siblings. There is no known family history of similar clinical features in the family. The affected individual’s clinical condition continued to deteriorate, requiring hospitalization for refractory seizures, mood disturbance and hallucinations, and dysphagia with aspiration pneumonia. She died while hospitalized.

In a consanguineous Saudi Arabian family (F9), a different missense variant in *MRPL49* at the same 88 residue (c.263G>A [GenBank: NM_004927.4] [p.Arg88His]) was identified in two brothers affected with developmental delay, SNHL, and ataxia. Profound hearing loss in the younger affected sibling (F5:II-2) was apparent after birth, with distortion product otoacoustic emissions absent at all tested frequencies bilaterally. Brainstem auditory evoked potentials were abnormal with prolonged peripheral latencies, poor waveform reproducibility, and poor proximal wave III/V with an increased threshold. He has never walked or crawled but sat unsupported at the age of 18 months. At age 6 years, he had significant cognitive delay and was only able to say two words. His OFC was 46 cm (−4.1 SD). He had raised plasma lactate on multiple occasions, with a maximal recorded level of 2.82 mmol/L (normal < 2). Throughout childhood, he has experienced recurrent episodes of hypoglycemia. A recent assessment of renal function demonstrated urea of 7.9 mmol/L (at the upper end of the N, 2.1–8.5), serum creatinine on the higher side at 50 μmol/L (range: 18–46), and raised serum potassium of 5.6 mmol/L (N: 3.6–5.2). The elder affected brother (F9:II-1) was more severely affected, with a similar pattern of profound hearing loss and significant developmental delay. Ophthalmological assessment was unremarkable. His lactate was raised at 2.30 mmol/L. At age 9 years, he had stage 3 chronic kidney disease of unknown etiology (eGFR: 51 mL/min/1.73 m^2^). Renal ultrasound revealed slightly echogenic kidneys with mild distension of the left renal collecting system. He had recurrent hyperkalemia, and a renal biopsy was consistent with chronic interstitial nephritis.

**Figure 2:**
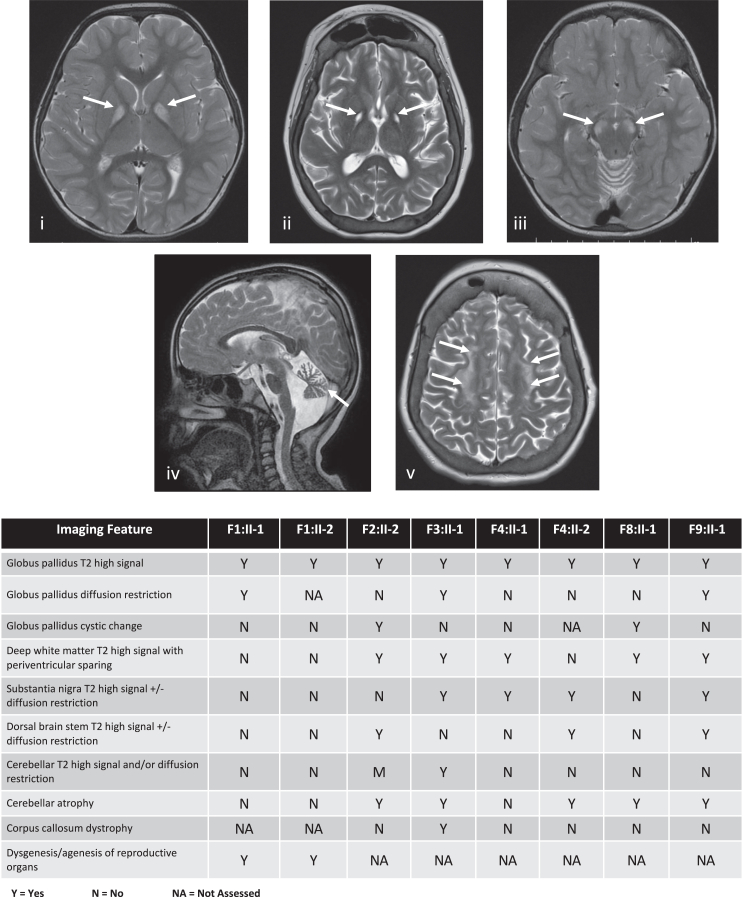
Selected brain MR images demonstrating features of individuals with variants in *MRPL49* and an accompanying summary of all imaging features shared across 8 individuals from 6 unrelated families All 8 affected individuals with available brain imaging had symmetrical high T2 signal changes in the globi pallidi (i). This feature progressed to symmetrical cystic changes in two affected individuals with more advanced disease (ii). In 3/8 affected individuals, there was symmetrical high T2 signals and diffusion restriction (not pictured) involving the brain stem, including the substantia nigra (iii) and the dorsal brainstem. In 5/8 affected individuals, there were symmetrical white matter high T2 signal changes, becoming more diffuse and confluent with more advanced disease. There was also evidence of cerebral atrophy in 5/8 affected individuals, particularly involving the cerebellum (iv) but also affecting cortical gray matter and sub-cortical and deep white matter in individuals with more advanced disease (ii and v). The chart summarizes the features noted on imaging of the affected individuals.

Brain magnetic resonance (MR) imaging was available from eight affected individuals from six families (Figures 2 and S3) as were brain imaging reports from three further affected individuals (F5:II-2, F6:II-1, and F7:II-1). This imaging demonstrates consistent progressive patterns with symmetrical involvement of the globi pallidi in all eight affected individuals, presenting as a high T2 signal with or without associated diffusion restriction or as cystic change. These features are consistent with the brain imaging reports from individuals F5:II-2, F6:II-1, and F7:II-1, in whom symmetrical signal increases were observed in the globus pallidi.

In five individuals, there is a symmetrical high T2 signal in the deep white matter (centrum semiovale) with periventricular sparing. Involvement of the brain stem is seen in three and cerebellar atrophy in five of the eight affected individuals. Globus pallidus cystic change, cerebellar atrophy, and deep white matter high T2 signal changes were associated with more advanced disease (Figure 2).

To summarize the clinical features of the affected individuals with *MRPL49* bi-allelic variants (Figures 1B and 2), where data were accessible, the majority of affected individuals have SNHL (9/12), POI in post-pubertal females (4/6), brain white matter changes (11/11), learning disability (12/13), and microcephaly (5/8). Additionally, some individuals experienced hypoglycemia and had evidence of renal or retinal disease. The different bi-allelic variants in *MRPL49* in multiple unrelated families with similar clinical features meet the evidence criteria for a rare disease-gene association.^17^ We proceeded to generate cellular and *in vitro* functional data to support this disease-gene association.

**Figure 3:**
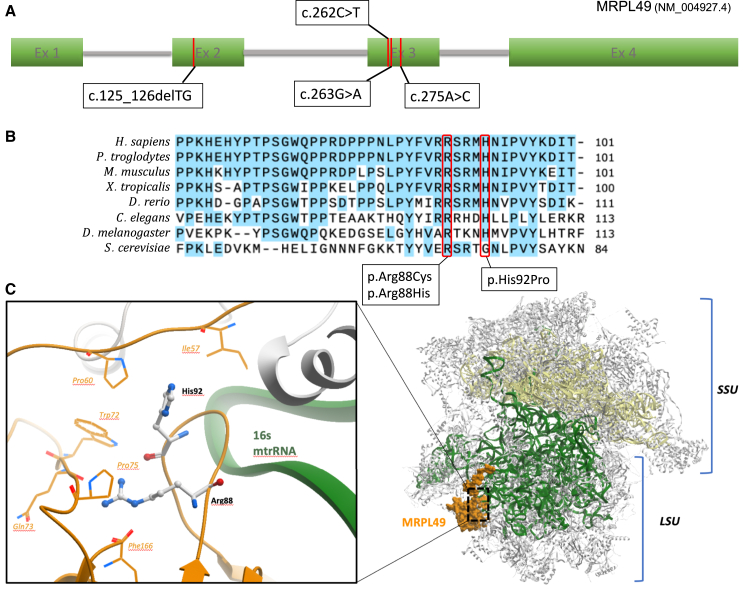
*In silico* modeling of MRPL49 variants (A) Schematic representation of *MRPL49* transcript and disease-associated variant locations. All three missense variants are clustered in exon 3, whereas the single frameshift variant is located in exon 2. (B) Evolutionary conservation of MRPL49 affected residues across a broad range of orthologous species. Conserved residues are colored blue, and variant amino acids (arginine 88 and histidine 92) are highlighted in red. (C) Three-dimensional representation of the location of MRPL49 (orange) within the human mitoribosome (PDB: 7QI4). Large (mt-LSU) and small (mt-SSU) units are colored in gray, 16S rRNA in green, and 12S rRNA in yellow.

The amino acids in MRPL49 at positions 88 and 92 are conserved across multiple species (Figure 3B). In gnomAD v.4.0, the variant alleles are reported at frequencies consistent with a rare monogenic disorder (Table S2); of note, p.His92Pro is represented as 22 of 86,250 alleles in a South Asian subset.^18^ No homozygous loss-of-function alleles are reported in gnomAD v.4.0, and *in silico* predictors indicated that the missense variants would have a deleterious effect on function (Table S3). Structurally, residues Arg88 and His92 are situated at a beta-hairpin loop of the MRPL49 protein, which forms part of the protein interface with 16S rRNA (Figure 3C). Arg88 is a strictly invariant amino acid among all orthologs, forming an important network of hydrogen bonds with nearby residues. His92 is 95% conserved among orthologs and packs against a hydrophobic core (Ile57, Pro60, and Trp72). The substitutions p.His92Pro (introducing a cyclic amino acid) as well as p.Arg88Cys and p.Arg88His (removing positive charge) are predicted to disturb the conformation of the MRPL49 beta-hairpin loop, which could impact the interaction with the 16S rRNA and the stability of the mt-LSU central protuberance.

**Figure 4:**
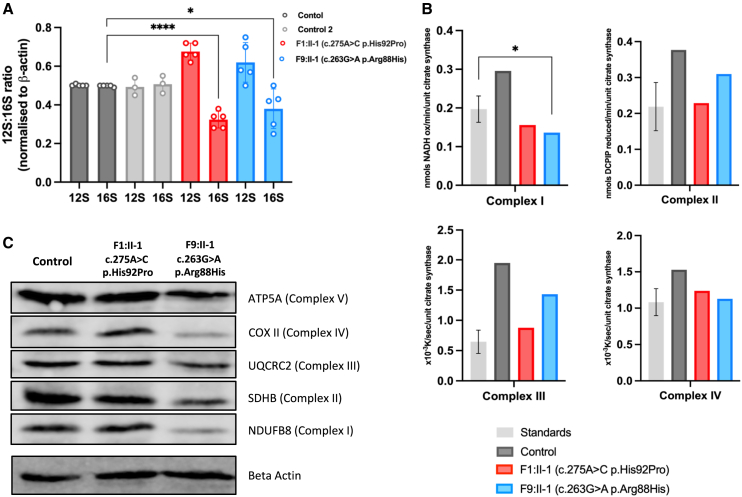
Functional and molecular assays of fibroblasts from affected individuals reveal significant reductions in levels and activities of mitochondrial respiratory chain complexes I and IV (A) MT-RNR1 (12S) and MT-RNR2 (16S) relative expression levels in fibroblasts from affected individuals and control individuals. Data are expressed as a ratio using relative expression to beta-actin. Error bars represent SD. ^∗∗∗∗^*p* < 0.0001 and ^∗^*p* = 0.0327; unpaired t test. (B) Mitochondrial respiratory chain enzyme activity assay in control (gray), F1:II-1 (red), and F9:II-1 (blue) fibroblasts. (C) Western blot analysis of proteins associated with each of the mitochondrial oxidative phosphorylation complexes in fibroblasts from F1:II-1, F9:II-1, and control individuals. Image is representative of three separate experiments.

To consider this predicted disturbed interaction, we measured the 12S:16S rRNA ratio in dermal fibroblasts available from affected individuals in F1 and F9. Previous studies investigating disease-associated variants in genes encoding proteins of the mt-LSU have consistently demonstrated a relative reduction in the levels of 16S rRNA.^10^^,^^19^^,^^20^ The levels of 16S rRNA were significantly reduced in the cells from both individuals with *MRPL49* variants compared to the 16S rRNA levels in control fibroblasts (*p* < 0.0001 and *p* = 0.0327; Figure 4A). These data demonstrate that the missense variants reduce 16S rRNA levels, consistent with the mechanism of other mt-LSU-associated disorders and may impact upon translation of mitochondrially encoded proteins. To examine mitochondrial protein translation, we measured the activity and levels of components of the OXPHOS complexes in fibroblasts from affected individuals compared to control fibroblasts. OXPHOS enzyme analysis^21^ revealed decreased activity of a complex I subunit in both affected individuals (Figure 4B). Western blot analysis revealed reduced steady-state levels of both complex I and complex IV subunits in fibroblasts from F9:II-1 but no significant changes in cells from F1:II-1. These differences reflect the sensitivity of the assays, as evidenced by the subsequent complexome profiling data, but the greater reductions in F9:II-1 are consistent with the more severe clinical phenotype in this affected individual.

**Figure 5:**
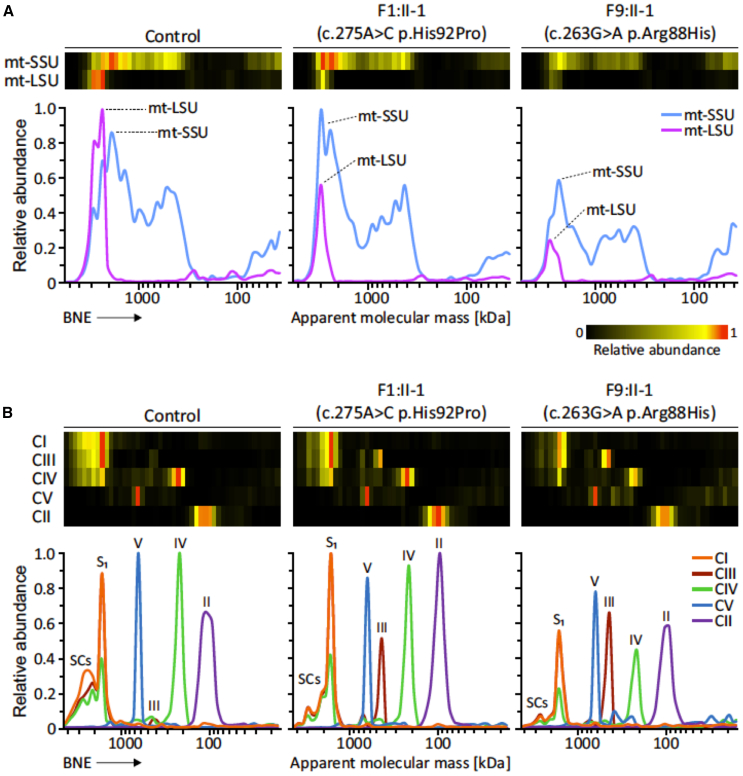
Complexome analysis of mitochondrial ribosomes and OXPHOS complexes from control and F1:II-1- and F9:II-1-derived fibroblasts Enriched mitochondria were solubilized with digitonin, separated by BNE, and analyzed by mass spectrometry (MS)-based complexome profiling. Protein abundance profiles of each mitoribosomal subunit and OXPHOS complex were generated by averaging the intensity-based absolute quantification (iBAQ) values of all their individual subunits identified by MS. Resultant profiles are illustrated as heatmaps and two-dimensional (2D) profile plots against the apparent molecular mass. (A) Abundance profiles of mitoribosomal small (mt-SSU) and large (mt-LSU) subunits showing a visible decrease in the fibroblasts from affected individuals. (B) Abundance profiles of the five mitochondrial OXPHOS complexes consistently showing a drop in their content, particularly for complexes I and IV. CI, CII, CIII, CIV, and CV stand for complexes I, II, III, IV, and V, respectively. S1, respiratory supercomplex formed by complexes I, III, and IV; SCs, higher-order respiratory complexes (respirasomes).

To explore more fully the effect of the *MRPL49* missense variants on OXPHOS complex assembly and the steady-state levels of other mitochondrial proteins, we performed complexome profiling of mitochondria from fibroblasts from affected individuals (Figure 5).^22^^,^^23^ Complexome profiling is a quantitative mass spectrometry (MS) approach previously used to characterize deficiencies of other respiratory complex genes.^24^^,^^25^^,^^26^ Enriched mitochondrial fractions from both affected individuals and control fibroblasts were subjected to blue native electrophoresis (BNE), systematic dissection of the polyacrylamide gel, tryptic digestion, and tandem MS, as reported previously.^27^^,^^28^ MS data, identification, quantification, and complete interaction profiles of mitochondrial proteins from individuals F1:II-1, F9:II-1, and control fibroblasts were deposited to the ProteomeXchange Consortium^29^ via the PRIDE partner repository^30^ with the dataset identifier PXD056347. The abundance of the proteins of interest in each discrete section of the BNE gel was visualized as heatmaps and line charts (Figure 5). The MS analysis identified all 30 protein components of the mt-SSU and 51 out of 52 protein components of the mt-LSU (Figure S4). A few components were expected to be found in their free forms due to their known labile interactions and poor stabilization during BNE.^31^ The complexome data reveal a general reduction in the content of mitoribosomes, as evidenced by lower levels of individual mt-LSU and mt-SSU in fibroblasts from both affected individuals compared to control individuals (Figure 5A). In F1:II-1, we observed decreases of ∼15% in mt-SSU and 60% in mt-LSU. In F9:II-1, the decrease was more pronounced, with reductions of ∼40% in mt-SSU and 70% in mt-LSU. Strikingly, variants in *MRPL49* did not change the migration patterns of any of the individual components (Figure S4), in particular those from the direct interactors, MRPL4, MRPL15, MRPL57, and MRPL64.^32^

The complexome data reflect a reduction in the assembled respiratory supercomplex S1 (I+III+IV) and larger respirasomes (SCs) alongside a pronounced reduction in complexes I and IV in fibroblasts from the affected individuals (Figure 5B). In F1:II-1 fibroblasts, complex I levels decreased by approximately 30%, while complex IV levels showed a 25% reduction. These alterations are more substantial in F9:II-1 fibroblasts, where complex I and complex IV levels dropped by ∼70% and 65%, respectively. Notably, there were ∼15% and ∼50% reductions in complex III-containing supercomplexes, with corresponding 7- and 11-fold increases in the accumulation of free-form complex III in F1:II-1 and F9:II-1, respectively. Complex V levels decreased by ∼20% in both affected individuals. Complex II levels were comparable to the control individual in F1:II-1 but were ∼15% lower in F9:II-1. Finally, no visible accumulation of assembly intermediates or major defects in OXPHOS biogenesis were observed (Figure S5).

Our cumulative data have determined that bi-allelic variants in *MRPL49* are associated with a complex variable mitochondrial phenotype, characterized by SNHL, POI, leukodystrophy, retinopathy, and learning disability. The complexome data detailing the marked deficiency of complexes I and IV in fibroblasts from affected individuals are consistent with a reduction in mitochondrial protein translation and define bi-allelic variants in *MRPL49* as a new cause of COXPD.

MRPL49 is one of a group of proteins within the mt-LSU that has no apparent homolog in bacterial, chloroplast, archaebacterial, or cytosolic ribosomes.^2^ However, it does have a homolog in yeast (Img2),^33^ which is required for mitochondrial genome integrity. The specific function of MRPL49 within the mitoribosome has not been determined, but it may compensate for lost rRNA and stabilize bypass segments within the mt-LSU.^3^ To date, no *mrpl49* knockout mouse models have been reported.^34^ We would predict that such a model would be non-viable. Apart from *Mrpl56* knockout mice,^35^ all other knockouts of *Mrpl* genes are non-viable.^34^ This prediction of lethality of an *MRPL49* knockout aligns with our data that the disease-associated variants are hypomorphic, evidenced by the reduced levels of MRPL49 in complexome profiling of fibroblasts from affected individuals.

To date, variants in over 50 genes have been associated with COXPD,^36^ with striking clinical variability of growth retardation, microcephaly, altered tone, leukodystrophy, cardiomyopathy, and liver dysfunction. It is notable that interfamilial phenotypic variability is present even in this small cohort of individuals with *MRPL49* variants. The two families with homozygous His92Pro variants have intrafamilial phenotypic concordance but striking interfamilial differences. The affected females in F1 have classical features of Perrault syndrome, whereas the affected female in F2 has no evidence of ovarian insufficiency and no hearing loss. We undertook immunofluorescence of sections from the ear of wild-type adult mice, as previously described,^37^ to determine if MRPL49 is present in cells of the inner ear. MRPL49 was observed in the mitochondria in the outer hair cells, inner hair cells, and supporting cells, consistent with a role in hearing function, but this pattern does not explain the variable hearing phenotype (Figure S2). We infer an undefined genetic modifier underpinning these phenotypic differences.

In contrast to other disorders associated with disruption of the mt-LSU, there was no evidence of cardiomyopathy or liver involvement in individuals with *MRPL49* variants. It will be important to ascertain additional affected individuals to determine the full phenotypic spectrum and the possibility of adult neurocognitive decline as seen in the individual F4 who died at age 37 years. However, it is notable that among all the affected individuals reported in this study, there is a consistent pattern of progressive leukodystrophy. This consistency contrasts with other genes associated with Perrault syndrome where brain white matter changes are variable in frequency, from commonly present in individuals with *CLPP*-associated Perrault syndrome to absent with *HARS2*-associated disease.^13^

It is notable that the affected individuals had variants altering residues 88 and 92 in MRPL49. These residues lie in a loop of MRPL49 that interacts with the 16S rRNA, and variants in this loop result in reduced levels of the 16S rRNA, with subsequent effects on mitochondrial protein translation. We would predict that other MRPL49 residues at this interface are likely to be associated with this pleiotropic phenotype. Complexome profiling demonstrated a reduction in levels of all mt-LSU protein components of the mitoribosome in the fibroblasts from affected individuals, which were more pronounced in F9:II-1 (Figures 5 and S4). A lower but visible drop was also observed in the mt-SSU content (Figures 5 and S4). Recent cryoelectron microscopy (cryo-EM) studies have indicated that MRPL49 interacts intricately with MRPL4, MRPL15, MRPL57, and MRPL64, alongside the 16S rRNA within the mt-LSU.^32^ Given that no changes in the migration patterns of these components were observed, defects at the assembly level seem unlikely. We propose that the two variants in *MRPL49* lead to a loss of the proper three-dimensional architecture of this critical region, resulting in the reduced stability of the entire subunit and potential degradation. Consequently, a lower content of fully assembled mitoribosomes likely impacts mitochondrial protein translation rates, diminishing the synthesis of OXPHOS subunits, especially for complex I and complex IV (Figure 5B). Together, these issues not only compromise the correct assembly of OXPHOS complexes but also result in a reduced yield of mitochondrial ATP synthesis, leading to an energy crisis at the cellular level.

In summary, we describe bi-allelic variants in *MRPL49* associated with a pleiotropic phenotype, including SNHL, POI, leukodystrophy, learning disability, and retinopathy in unrelated families. This work expands the number of genes known to cause a phenotype consistent with both Perrault syndrome and COXPD. Further, it increases the known number of genes encoding components of the mt-LSU that are associated with human disease and provides insights into the function of the mitoribosome in the control of mitochondrial homeostasis.

## Data and code availability

• The *MRPL49* variants were submitted to ClinVar (https://www.ncbi.nlm.nih.gov/clinvar/) (GenBank: NM_004927.4; accession numbers SCV004242144–SCV004242147). The exome datasets supporting this study have not been deposited in a public repository because of ethical restrictions but are available from the corresponding author upon request.
• The mass spectrometry data have been deposited to the ProteomeXchange Consortium via the PRIDE partner repository with the dataset identifier PXD056347 and 10.6019/PXD056347.

## Acknowledgments

We thank the families for their participation. This study was supported by the 10.13039/501100000265Medical Research Council (MR/W019027/1 RTO, R.W.T. and W.G.N.); 10.13039/501100000703Action on Hearing Loss (S35 and S60_Newman); 10.13039/501100000317Action Medical Research (GN2494); NIH-10.13039/100000071National Institute of Child Health and Human Development (NICHD) (R01HD109342 to S.M.L. and I.S.); 10.13039/100014653NIHR Manchester Biomedical Research Centre (IS-BRC-1215-20007 and NIHR203308); the 10.13039/501100013372Wellcome Trust Centre for Mitochondrial Research (203105/Z/16/Z to R.W.T.); the UK NHS Highly Specialised “Rare Mitochondrial Disorders of Adults and Children” Service (R.W.T.); the 10.13039/501100022186Lily Foundation (R.W.T.); and 10.13039/100000055NIDCD/NIH (R01DC012564 to Z.M.A.). The research team acknowledges the support of the National Institute for Health Research through the Comprehensive Clinical Research Network. I.W. is supported by the 10.13039/501100001659Deutsche Forschungsgemeinschaft (DFG): SFB1531-S01, project no. 456687919; TRR267/Z2, project no. 403584255; and WI 3728/3-1, project no. 515944830. We would like to thank Jana Meisterknecht for her excellent technical support.

## Declaration of interests

The authors declare no competing interests.

## Web resources

dbSNP, https://www.ncbi.nlm.nih.gov/projects/SNP/
Exome Variant Server, http://evs.gs.washington.edu/EVS/
GenBank, https://www.ncbi.nlm.nih.gov/genbank/
GeneMatcher, https://genematcher.org/
Ensembl – Variant Effect Predictor, https://www.ensembl.org/Tools/VEP.
Clustal – Omega, https://www.ebi.ac.uk/jdispatcher/msa/clustalo.
GTEx, https://gtexportal.org/home/
gnomAD, http://gnomad.broadinstitute.org/
FoldX, http://foldxsuite.crg.eu/
LOVD, https://www.lovd.nl/
OMIM, https://www.omim.org/
MutationTaster, http://www.mutationtaster.org/
PDB, https://www.rcsb.org
PolyPhen-2, http://genetics.bwh.harvard.edu/pph2/
ProteomeXchange Consortium https://www.proteomexchange.org.
SIFT, http://sift.bii.a-star.edu.sg

## Supplemental information

Document S1. Figures S1–S5, Tables S1–S4, and supplemental methods

Document S2. Article plus supplemental information
